# The effects of comorbid Tourette symptoms on distress caused by compulsive-like behavior in very young children: a cross-sectional study

**DOI:** 10.1186/s13034-019-0290-3

**Published:** 2019-06-28

**Authors:** Ryunosuke Goto, Miyuki Fujio, Natsumi Matsuda, Mayu Fujiwara, Marina Nobuyoshi, Maiko Nonaka, Toshiaki Kono, Masaki Kojima, Norbert Skokauskas, Yukiko Kano

**Affiliations:** 1 The University of Tokyo Hospital, 7-3-1 Hongo, Bunkyo-ku, Tokyo, 113-8655 Japan; 2 Graduate School of Education, The University of Tokyo, 7-3-1 Hongo, Bunkyo-ku, Tokyo, 113-8655 Japan; 3 Department of Child Neuropsychiatry, Graduate School of Medicine, The University of Tokyo, 7-3-1 Hongo, Bunkyo-ku, Tokyo, 113-8655 Japan; 4 Department of Child Psychiatry, The University of Tokyo Hospital, 7-3-1 Hongo, Bunkyo-ku, Tokyo, 113-8655 Japan; 5 Department of Community Mental Health and Law, National Institute of Mental Health, National Center of Neurology and Psychiatry, 4-1-1 Ogawahigashi, Kodaira, Tokyo, 187-8553 Japan; 6 Regional Centre for Children and Youth Mental Health and Child Welfare-Central Norway, Norwegian University of Science and Technology, RKBU Midt-Norge, NTNU, Postboks 8905 MTFS, 7491 Trondheim, Norway

**Keywords:** Preschool children, Tourette’s disorder, Compulsive-like behavior, Distress, Comorbidity, Tics

## Abstract

**Background:**

Many children 4 to 6 years old exhibit compulsive-like behavior, often with comorbid Tourette symptoms, making this age group critical for investigating the effects of having comorbid Tourette symptoms with compulsive-like behavior. However, these effects have not yet been elucidated: it is unclear whether having comorbid tics with compulsive-like behavior leads to lower quality of life. This cross-sectional study aims to investigate the effect of comorbid Tourette symptoms on distress caused by compulsive-like behavior in very young children.

**Methods:**

Self-administered questionnaires were distributed to guardians of children aged 4 to 6 attending any of the 59 public preschools in a certain ward in Tokyo, Japan. The questionnaire contained questions on the presence of Tourette symptoms, the presence of specific motor and vocal tics, frequency/intensity of compulsive-like behavior, and the distress caused by compulsive-like behavior, which was rated on a scale of 1 to 5. Additionally, questions on autism spectrum disorder (ASD) traits, attention-deficit/hyperactivity disorder (ADHD) traits, internalizing behavior traits, and externalizing behavior traits were included in the questionnaire as possible confounders of distress caused by compulsive-like behavior. Wilcoxon rank-sum tests were conducted to compare the distress caused by compulsive-like behavior and frequency/intensity of compulsive-like behavior between children in the Tourette symptoms group and the non-Tourette symptoms group. Furthermore, a stepwise regression analysis was performed to assess the effects of the independent variables on distress caused by compulsive-like behavior. Another stepwise regression analysis was performed to assess the relationship between distress caused by compulsive-like behavior and the presence of five specific motor and vocal tics.

**Results:**

Of the 675 eligible participants, distress due to compulsive-like behavior was significantly higher in children in the Tourette symptoms group compared to the non-Tourette symptoms group (2.00 vs 1.00, P < 0.001). Stepwise regression analysis showed that frequency/intensity of compulsive-like behavior, being in the Tourette symptoms group, ASD traits, and internalizing behavior traits were predictors of distress due to compulsive-like behavior. Two specific tics, repetitive noises and sounds and repetitive neck, shoulder, or trunk movements, were significant predictors of distress due to compulsive-like behavior.

**Conclusions:**

Comorbid Tourette symptoms may worsen distress caused by compulsive-like behavior in children 4 to 6 years old, and specific motor and vocal tics may lead to greater distress.

## Background

Compulsivity is common among very young children, with more than 75% of 2- to 4-year-old children exhibiting compulsive-like behavior [1]. Some of these children receive the diagnosis of obsessive–compulsive disorder (OCD), a common and long-lasting disorder characterized by uncontrollable, reoccurring thoughts (obsessions) and behaviors (compulsions) for which he or she feels the urge to repeat over and over [1]. Tourette’s disorder is another relatively common disorder characterized by motor and vocal tics present for more than 1 year. Tourette’s disorder is found in 0.60% of 7- to 9-year-old children [2]. Children with OCD often present with comorbid psychiatric conditions such as mood disorders, psychosis, anxiety disorders, and neurological diseases [3]. Notably, studies have shown that 20–38% of children with OCD also have tics [4–8].

Studies have focused on the adverse effects of comorbid OCD on Tourette’s disorder. It has been shown that Tourette’s disorder patients with OCD symptoms have lower global functioning scores [9]. Additionally, the psychosocial quality of life is significantly lower in children, adolescents, and adults with Tourette’s disorder and OCD compared to those with Tourette’s disorder only [10–12].

However, the effects of comorbid Tourette symptoms on compulsive-like behavior have not been elucidated in very young children. Although patients with very early onset OCD have higher rates of comorbid Tourette’s disorder and more psychosocial difficulties [13], it is unclear if having comorbid Tourette symptoms with compulsive-like behavior leads to lower quality of life in very young children.

Compulsive-like behavior is generally found in more than 75% of children 2 to 4 years old and decreases until 6 years of age, while the onset of tics is reported to be typically 4 to 6 years old [1, 12, 14, 15]. Therefore, 4 to 6 years of age seems to be a critical age range for investigating the effects of having comorbid Tourette symptoms with compulsive-like behavior.

The present study aims to elucidate whether having comorbid Tourette symptoms with compulsive-like behavior worsens distress due to compulsive-like behavior in children 4 to 6 years of age.

## Methods

### Study design and procedure

We conducted a cross-sectional study to determine whether the presence of comorbid tics impacts distress caused by compulsive-like behavior in preschool children. Only guardians for whom written informed consent were obtained were included in the study, and the study was approved by the ethics committee of the University of Tokyo (IRB number: 11316).

### Participant enrollment

In this study, self-administered questionnaires were distributed to guardians of children in their 2nd or 3rd year in preschool, aged 4 to 6.

The questionnaires were first distributed to the principal of each of the 59 public preschools in a certain ward with a population of about 700,000 people in Tokyo, Japan, which provide care and education for infants and children up to 6 years old before the child enters elementary school. The questionnaires were then distributed to the parents or guardians of children attending the preschool who were 4 or 5 years old at the beginning of the 2017 school year. The guardians were asked to take the questionnaire home, fill out the questionnaire, and mail the questionnaire to the address provided if they agreed to participate in the study. Guardians who could not read or write in Japanese were excluded from the study.

### Assessment tools and variables

The questionnaires were administered in Japanese. In certain parts of the questionnaires the original questions were written in English, in which case they were translated into Japanese by a group of clinicians with extensive knowledge and experience in the field of child psychiatry.

The presence of Tourette symptoms was assessed using seven questions derived and translated from questions on Tourette’s disorder and chronic tics used in Avon Longitudinal Study of Parents and Children Cohort (ALSPAC), which have been utilized in a previous study that assessed Tourette symptoms with a Japanese questionnaire [16, 17]. Six questions were utilized directly from the original questionnaire, while one question was added to investigate whether the tic(s) were present more than a year ago to determine the chronicity of the tic(s). Among the original six were three questions on motor tics (Q1: In the past year, has your child had any repeated movements of parts of the face and head?; Q2: In the past year, has your child had repeated movements of the neck, shoulder, or trunk?; Q3: In the past year, has your child had repeated movements of arms, hands, legs, or feet?), two on vocal tics (Q4: In the past year, has your child had repeated noises and sounds, such as coughing, clearing throat, grunting, gurgling, and hissing?; Q5: In the past year, has your child had repeated words or phrases?) and one on the frequency of the tic(s). For all the questions except the one on frequency of the tics, the participant was asked to choose from “definitely”, “probably”, and “not at all” present. The participants were asked to choose the frequency from “less than once a month”, “once to three times a month”, “once a week”, “more than once a week”, and “everyday”. Three definitions of Tourette’s disorder, based on diagnostic stringency, were used in the original ALSPAC study: narrow, intermediate, and broad. Of these, the authors determined that the narrow and intermediate definitions were suitable because the rates of Tourette’s disorder according to the narrow and intermediate definitions were consistent with those of previous studies [16]. In the present study, both the narrow and intermediate definitions were used for the analyses. As an exception, the intermediate definition was used for the subgroup analysis because only 17 out of over 700 participants met the narrow definition, a number not suitable for subgroup analysis. We define children that meet these definitions as being in the Tourette symptoms group instead of Tourette’s disorder group, since it is not appropriate to determine that a child has Tourette’s disorder solely based on a questionnaire by the guardian. The intermediate definition is as follows:

*Answered “definitely present” or “probably present” to motor tics AND vocal tics*

*AND*

*Frequency is “every day” or “more than once a week”*

*AND*

*Answered “definitely” or “probably” to whether tics existed more than one year ago*

Subjects with only repetitive movements of the arms, hands, legs, or feet or with only repetitive words or phrases were classified into the non-Tourette symptoms group to exclude non-tic movements such as stereotypy or isolated echolalia, just as it was done in the ALSPAC study. The same criteria have been used for another ALSPAC study, adding to the validity of this definition [18].

Independent variables included the child’s age, gender, being in the Tourette symptoms group, and frequency/intensity of compulsive-like behavior. The frequency/intensity of compulsive-like behavior was assessed with the original childhood routines inventory (CRI) score, a criterion used to evaluate compulsive-like behavior in young children [1]. The CRI score has been used in many studies including a study on compulsive-like behavior in Japan [1, 19–21]. In addition, autism spectrum disorder (ASD) traits, attention-deficit/hyperactivity disorder (ADHD) traits, internalizing behavior traits, and externalizing behavior traits were included as independent variables because these were expected to be possible confounders of distress due to compulsive-like behavior, given the pervasiveness of comorbid psychiatric disorders in children [3]. Questions on ASD, ADHD, internalizing behavior, and externalizing behavior traits were created specifically for this study by experts in child and adolescent health; each trait was assessed in two to three original questions, which asked for the frequency of behaviors related to each trait. These questions were created to capture the main components of each trait based on the Diagnostic and Statistical Manual of Mental Disorders (DSM-5), the Autism Spectrum Quotient: Children’s Version (AQ -Child), the ADHD Rating Scale (ADHD-RS), and Child Behavior Checklist (CBCL), and were purposefully designed to be concise for feasibility [15, 22–24].

The outcome was distress caused by compulsive-like behavior, and the participant was asked the following question after being asked about the presence of individual compulsive-like behaviors: “does your child seem distressed if he/she does not perform any of the above behaviors?” The participant was asked to rate the degree of distress from 1 (never distressed) to 5 (always distressed). Whereas in Evans’s original study the distress caused by each compulsive-like behavior was examined, our study assessed the overall distress caused by all of the compulsive-like behavior combined [1].

The relationship between the independent variables and the distress caused by compulsive-like behavior was examined.

### Data analysis

The distress due to compulsive-like behavior and the CRI score were compared between the Tourette symptoms group and non-Tourette symptoms group using the Wilcoxon rank-sum test.

A stepwise ordinal logistic regression analysis was then performed to assess the relationship between distress caused by compulsive-like behavior and being in the Tourette symptoms group, CRI score, ASD traits, ADHD traits, internalizing behavior traits, externalizing behavior traits, and the participants’ age and gender. The CRI score was included as a measure of the frequency/intensity of compulsive-like behavior, which could worsen the distress. Furthermore, the participants’ age, gender, ASD traits, ADHD traits, internalizing behavior traits, and externalizing behavior traits were also used as independent variables.

Among those who were included in the Tourette symptoms group, another stepwise ordinal logistic regression analysis was performed to assess the relationship between distress caused by compulsive-like behavior and the presence of each of the five types of tics (face and head; neck, shoulder, or trunk; arms, hands, legs, or feet; noises and sounds; repeated words or phrases), along with participants’ age, gender, ASD, ADHD, internalizing behavior, and externalizing behavior traits. Tics for which the response was “probably” or “definitely” present were considered present.

Only responders whose answers were available for all variables in the statistical analyses were included, and all statistical analyses were performed using Stata SE 14. The significance level was set at P < 0.05.

## Results

Of the 2,592 questionnaires that were distributed, 776 were collected (response rate = 29.9%). The total number of responses included in the Wilcoxon rank-sum tests and the first ordinal logistic regression analysis (Tables 1, 2, 3, 4) was 675 (all responses with missing answers were excluded from the analysis). The second ordinal logistic regression analysis (Table 5) was performed on 69 children who met the intermediate definition of Tourette symptoms.

**Table 1:** Group differences of CRI score and distress due to compulsive-like behavior for the intermediate definition

Item	Tourette symptoms group n = 69		Non-Tourette symptoms group n = 606		P-value
	Median	IQR	Median	IQR	
CRI score	2.21	(1.95–2.89)	1.74	(1.42–2.21)	< 0.001
Distress due to compulsive-like behavior	2.00	(1.00–3.00)	1.00	(1.00–1.00)	< 0.001

P-values are from Wilcoxon rank-sum tests for Tourette symptoms group vs. non-Tourette symptoms group. Both CRI score and distress due to compulsive-like behavior were found to be significantly different between groups

**Table 2:** Group differences of CRI score and distress due to compulsive-like behavior for the narrow definition

Item	Tourette symptoms group n = 16		Non-Tourette symptoms group n = 659		P-value
	Median	IQR	Median	IQR	
CRI score	2.11	(1.76–2.26)	1.79	(1.42–2.26)	0.072
Distress due to compulsive-like behavior	2.00	(1.00–3.50)	1.00	(1.00–2.00)	< 0.001

P-values are from Wilcoxon rank-sum tests for Tourette symptoms group vs. non-Tourette symptoms group. Distress due to compulsive-like behavior were found to be significantly different between groups

**Table 3:** Stepwise ordinal logistic regression analysis on 675 children

Predictors	All eligible participants n = 675		
	OR	95% CI	P-value
Tourette symptoms (intermediate)	2.46	(1.45–4.16)	0.001
CRI score	5.06	(3.62–7.08)	< 0.001
ASD traits	1.27	(1.05–1.54)	0.013
Internalizing behavior traits	1.28	(1.06–1.55)	0.010

Distress caused by compulsive-like behavior was the dependent variable. The presence of Tourette symptoms (intermediate), CRI score, ASD traits, ADHD traits, internalizing behavior traits, externalizing behavior traits, and the participants’ age and gender were independent variables. All independent variables with P ≤ 0.05 were included in the model. The model’s P < 0.001 and Pseudo R^2^ = 0.1861

**Table 4:** Stepwise ordinal logistic regression analysis on 675 children

Predictors	All eligible participants n = 675		
	OR	95% CI	P-value
Tourette symptoms (narrow)	5.17	(1.97–13.55)	0.001
CRI score	5.47	(3.91–7.65)	< 0.001
ASD traits	1.35	(1.12–1.63)	0.002
Internalizing behavior traits	1.26	(1.05–1.53)	0.015

Distress caused by compulsive-like behavior was the dependent variable. The presence of Tourette symptoms (narrow), CRI score, ASD traits, ADHD traits, internalizing behavior traits, externalizing behavior traits, and the participants’ age and gender were independent variables. All independent variables with P ≤ 0.05 were included in the model. The model’s P < 0.001 and Pseudo R^2^ = 0.1855

**Table 5:** Stepwise ordinal logistic regression analysis on children who met the intermediate definition of Tourette symptoms

Predictors	Tourette symptoms group n = 69		
	OR	95% CI	P-value
Age	0.36	(0.16–0.78)	0.010
Neck, shoulder, or trunk movement	3.11	(1.11–8.71)	0.015
Noises and sounds	7.37	(1.50–36.11)	0.014
CRI score	8.96	(3.52–22.85)	< 0.001
ASD traits	1.63	(1.10–2.41)	0.015

Distress caused by compulsive-like behavior was the dependent variable. The presence of each of the five types of tics (face and head; neck, shoulder, or trunk; arms, hands, legs, or feet; noises and sounds; repeated words or phrases), ASD traits, ADHD traits, internalizing behavior traits, externalizing behavior traits, and the participants’ age and gender were independent variables. Tics for which the response was “probably” or “definitely” present were considered present. All variables with P ≤ 0.05 were included in the model. The model’s P < 0.001 and Pseudo R^2^ = 0.2450

Among the children, there were 404 males and 357 females. The average age was 5.25 (SD = 0.66).

The Wilcoxon rank-sum tests showed that CRI scores (2.21 vs 1.74, P < 0.001) and distress caused by compulsive-like behavior (2.00 vs 1.00, P < 0.001) was significantly higher in the Tourette symptoms group (n = 69) compared to the non-Tourette symptoms group (n = 606) (Table 1) for the intermediate definition. Only distress caused by compulsive-like behavior (2.00 vs 1.00, P < 0.001) was significantly higher in the Tourette symptoms group (n = 16) compared to the non-Tourette symptoms group (n = 659) (Table 2). A stepwise ordinal logistic regression analysis showed that frequency/intensity of compulsive-like behavior, measured with CRI score, and the presence of Tourette symptoms, ASD traits, and internalizing behavior traits were significant predictors of distress due to compulsive-like behavior, for both the narrow and intermediate definitions of Tourette symptoms (Table 3, P < 0.001, Pseudo R^2^ = 0.1861; Table 4, P < 0.001, Pseudo R^2^ = 0.1855). Among children who met the criteria for the intermediate definition of Tourette symptoms, a stepwise ordinal logistic regression analysis revealed that CRI score, age, and the presence of ASD traits, repetitive noises and sounds, and repetitive neck, shoulder, or trunk movements were significant predictors of higher distress due to compulsive-like behavior (Table 5, P < 0.001, Pseudo R^2^ = 0.2450).

## Discussion

### Principal findings

The present study is the first to analyze the adverse effects of Tourette symptoms on compulsive-like behavior in very young children [10–12].

In very young children, being in the Tourette symptoms group was found to be associated with greater distress due to compulsive-like behavior. This implies that when tics are present in a compulsive very young child, it increases the risk for greater distress and may necessitate careful follow-up.

Our results also show that if a child with comorbid tics and compulsive-like behavior repeats noises and sounds or repeats movements of the neck, shoulder, or trunk, the child tends to be more distressed. The presence of these tics in a child with compulsive-like behavior may be an indicator that the child requires specialized monitoring and intervention in the future. Whether specific types of tics can worsen distress in a child has not been investigated before, and our findings warrant further investigation in future studies.

Furthermore, ASD and internalizing behavior traits but not ADHD or externalizing behavior traits were significantly associated with greater distress due to compulsive-like behavior. The co-occurrence of these traits with compulsive-like behavior too may be a sign of a necessity of careful follow-up.

### Strengths and limitations of this study

Distress is the main interest of the current study because of the following reasons: (1) distressed children obviously need more support and (2) distress may be an adequate measure of the pathological effects of compulsive-like behavior, given the expansion of the definition of OCD to include those with poor/absent insight in DSM-5 [15]. In terms of the latter, the new definition potentially adds a large number of children who lack or have very little symptom insight. Additionally, measuring insight in very young children is challenging, as suggested by the lack of studies on insight in OCD children younger than 6 years of age [25–27]. Consequently, it may not be appropriate to regard insight as an indicator of the presence of OCD; a potent alternative could be distress, a keyword repeatedly mentioned in DSM-5, although additional studies are needed to clarify the psychopathology [15].

The present study has several limitations. Though the Tourette symptoms group is defined in this study in accordance to previous studies, the proportion of children in the Tourette symptoms group in our study (10.2% for the intermediate definition and 2.4% for the narrow definition) is higher compared to previous reports. For instance, the proportion of children that meet the intermediate definition of Tourette’s disorder was reported to be 0.7% in a previous study [16]. This could be because the chronicity of Tourette symptoms was evaluated based on the self-judgement of the guardians (i.e. whether or not the tics were present more than a year ago) at one point in time, whereas in the original study chronicity was evaluated by asking tic screening questions at two different points in time [16].

Another limitation is that the questionnaires are not first-hand. The present study investigated whether the guardian who answered the questionnaire felt that the child seemed distressed, which cannot rule out the possibility that the child was not actually distressed. Additionally, parents who were distressed may have overestimated their children’s distress. However, we considered that getting first-hand information from preschool children would not be easy because of immaturity, and alternatively asked the guardians. Furthermore, the presence of Tourette symptoms was assessed based on observations by the guardians. This is a major limitation since the assessment of Tourette symptoms can be a challenge even for experienced clinicians, but it would have been impractical for clinicians to screen all children in such a large sample for the presence of individual Tourette symptoms. The same are true for the assessment of other items assessed in this study, such as compulsive-like behaviors and ASD, ADHD, internalizing behavior, and externalizing behavior traits. We determined that asking guardians, who look after the children on a daily basis, for the presence of these symptoms and traits was the best feasible alternative. Moreover, the questionnaires were collected via mail, meaning that guardians of children with more obvious symptoms or traits or who seemed more distressed by compulsive-like behaviors may have been more prone to sending in the questionnaires.

Though the logistic regression analysis does show the association between the presence of Tourette symptoms and distress due to compulsive-like behavior, it does not rule out the possibility that the association between Tourette symptoms and the distress due to compulsive-like behavior is influenced by confounding factors. In addition, the second regression analysis on children in the Tourette symptoms group had a relatively small sample size for performing a logistic regression analysis.

The results regarding ASD, ADHD, internalizing behavior, and externalizing behavior traits should be interpreted with more caution, as the questions regarding these traits are not well-validated, though they were derived from well-trusted sources such as DSM-5, AQ for children, ADHD-RS, and CBCL. It should be noted that the questions were simplified only to evaluate the traits, not to diagnose. Therefore, these results should only be used for reference and as indicators of possible confounding. Further investigation will be needed to confirm the association between compulsive-like behavior and ASD/internalizing behavior traits.

### Implications and future studies

Compulsive-like behavior is said to be most prevalent in 2- to 4-year-old children, while the onset of tics is most typically between 4 to 6 years of age [1, 15]. Given this, children 4 to 6 years old with compulsive-like behavior should be carefully monitored for comorbid Tourette symptoms, which could worsen the distress caused by the already present compulsive-like behavior. Furthermore, once tics are apparent, children should be monitored closely for any compulsive-like behavior to minimize the possible worsening effect of tics on distress due to compulsive-like behavior.

Future studies should focus on time-dependent relationships between the presence of Tourette symptoms and compulsive-like behavior. A longitudinal study is necessary in investigating whether having co-occurring Tourette symptoms and compulsive-like behavior leads to greater distress due to compulsive-like behavior or development of OCD in the long run. If the results are replicated, young children with co-occurring tics and compulsive-like behavior should be assigned to specialized care as high-risk patients. A longitudinal study should also investigate whether the presence of specific tics in a child with compulsive-like behavior worsens distress caused by compulsive-like behavior, which could reveal prognostic factors in children with comorbid Tourette symptoms and compulsive-like behavior.

## Conclusions

Four- to six-year-old children with Tourette symptoms tend to experience more distress due to compulsive-like behavior.

## Data Availability

The datasets used and analyzed during the current study are available from the corresponding author on reasonable request.

## Abbreviations

OCD: obsessive–compulsive disorder
ALSPAC: Avon Longitudinal Study of Parents and Children Cohort
CRI: childhood routines inventory
ASD: autism spectrum disorder
ADHD: attention-deficit/hyperactivity disorder
DSM-5: Diagnostic and Statistical Manual of Mental Disorders
AQ-Child: Autism Spectrum Quotient: Children’s Version
ADHD-RS: ADHD Rating Scale
CBCL: Child Behavior Checklist
